# Solutions to the nonlinear Schrödinger systems involving the fractional Laplacian

**DOI:** 10.1186/s13660-018-1874-9

**Published:** 2018-10-29

**Authors:** Meng Qu, Liu Yang

**Affiliations:** School of Mathematics and Statistics, Anhui Normal University, Wuhu, P.R. China

**Keywords:** 35J45, 35J60, 45G05, Fractional Laplacian, Nonlinear Schrödinger system, Moving plane method, Radial symmetry

## Abstract

In this paper, we consider the following nonlinear Schrödinger system involving the fractional Laplacian operator:

$$ \textstyle\begin{cases} (-\Delta)^{\frac{\alpha}{2}}u+au=f(v), \\ (-\Delta)^{\frac{\beta}{2}}v+bv=g(u), \end{cases}\displaystyle \quad \text{on } \Omega\subseteqq \mathbb{R}^{n}, $$

where $a,b\geq0$. When Ω is the unit ball or $\mathbb{R}^{n}$, we prove that the solutions $(u,v)$ are radially symmetric and decreasing. When Ω is the parabolic domain on $\mathbb{R}^{n}$, we prove that the solutions $(u,v)$ are increasing. Furthermore, if Ω is the $\mathbb{R}^{n}_{+}$, then we also derive the nonexistence of positive solutions to the system on the half-space. We assume that the nonlinear terms *f*, *g* and the solutions *u*, *v* satisfy some amenable conditions in different cases.

## Introduction

This paper is mainly devoted to investigating the properties of the solutions of the following system involving the fractional Laplacian operators:

1.1 $$ \textstyle\begin{cases} (-\Delta)^{\frac{\alpha}{2}}u+au=f(v), \\ (-\Delta)^{\frac{\beta}{2}}v+bv=g(u), \end{cases}\displaystyle \quad \mbox{for some } a,b\geq0, $$

with

$$ (-\Delta)^{\frac{\alpha}{2}}u(x)= C_{n,\alpha} \operatorname{P.V.} \int _{\mathbb {R}^{n}} \frac{u(x)-u(y)}{|x-y|^{n+\alpha}} \,dy $$

and

$$ (-\Delta)^{\frac{\beta}{2}}v(x)= C_{n,\beta} \operatorname{P.V.} \int _{\mathbb {R}^{n}} \frac{v(x)-v(y)}{|x-y|^{n+\beta}} \,dy, $$

where P.V. stands for the Cauchy principle value, $C_{n,\alpha },C_{n,\beta}>0$ and $0<\alpha, \beta<2$. To make sense for the integrals, we require $u\in C_{\mathrm {loc}}^{1,1} \cap L_{\alpha}$, $v\in C_{\mathrm{loc}}^{1,1} \cap L_{\beta}$, where

$$L_{\alpha} = \biggl\{ u \in L^{1}_{\mathrm{loc}}\bigl(\mathbb {R}^{n} \bigr) \Bigm| \int _{\mathbb{R}^{n}} \frac{|u(x)|}{1+|x|^{n+\alpha}} \, d x < \infty\biggr\} $$

and

$$L_{\beta} = \biggl\{ v \in L^{1}_{\mathrm{loc}}\bigl(\mathbb {R}^{n} \bigr) \Bigm| \int _{\mathbb {R}^{n}} \frac{|v(x)|}{1+|x|^{n+\beta}}\, d x < \infty\biggr\} . $$

For more background on the fractional Laplacian operator $(-\Delta )^{\frac{\alpha}{2}}$, we refer to [1–4]. We mention that there are also several applications involving the fractional Laplacian in mathematical physics [5–8], finance [9], image processing [10], and so on.

Since the fractional Laplacian is nonlocal, that is, it does not act by pointwise differentiation but as a global integral with respect to a singular kernel, this is the main difficulty in studying problems involving it. To circumvent this difficulty, Caffarelli and Silvestre [11] introduced the *extension method* (CS extension) to overcome the difficulty of nonlocality. Their idea is to localize the fractional Laplacian by constructing a Dirichlet to Neumann operator of a degenerate elliptic equation. We can also use the *integral equation method*, the *method of moving planes in integral forms*, and *regularity lifting* to investigate equations involving the fractional Laplacian. Recently, Chen, Li, and Li [12] developed a new method that can handle directly these nonlocal operators. They used this property to develop some techniques needed in the direct method of moving planes in the whole space $\mathbb{R}^{n}$ and the upper half-space $\mathbb{R}^{n}_{+}$, such as the narrow region principle and decay at infinity. The direct method of moving planes is very useful, and a series of fruitful results have been obtained. For more articles concerning the method of moving planes for nonlocal equations and systems, mainly for integral equations, we refer to [13–21].

In this paper, following the ideas of [12], among others, we consider the properties of the solutions to system (1.1) for different domains Ω. More precisely, we get the following four theorems. Firstly, we consider the case where Ω is the unit ball. For simplicity, we denote $B=B_{1}(0)$. We have

### Theorem 1.1

*Let* $u\in C(\overline{B})\cap\mathcal {C}_{\mathrm{loc}}^{1,1}(B)$ *and* $v\in C(\overline{B})\cap\mathcal {C}_{\mathrm{loc}}^{1,1}(B)$ *be positive solutions of the system*

1.2 $$ \textstyle\begin{cases} (-\Delta)^{\frac{\alpha}{2}}u+au=f(v),\quad {x}\in B, \\ (-\Delta)^{\frac{\beta}{2}}v+bv=g(u),\quad {x}\in B, \\ u=v=0,\quad {x}\notin B, \end{cases}\displaystyle \quad \textit{for some } a,b\geq0. $$

*with* $M>f'(\cdot)$, $g'(\cdot)>0$, *where* *M* *is a positive constant*. *Then* *u* *is radially symmetric and decreasing about the origin*.

### Remark 1.1

Li [22] considered the similar problem with $f(v)=v^{p}$ and $g(u)=u^{p}$. So Theorem 1.1 can be regarded as an extension of the result in [22].

We denote by

$$\Omega=\bigl\{ x=\bigl(x',x_{n}\bigr)\in \mathbb{R}^{n}\mid x_{n}> \bigl\vert x' \bigr\vert ^{2},x'=(x_{1},x_{2},\ldots ,x_{n-1})\bigr\} $$

the parabolic domain on $\mathbb{R}^{n}$.

### Theorem 1.2

*Let* $u\in L_{\alpha}(\mathbb{R}^{n})\cap\mathcal {C}_{\mathrm {loc}}^{1,1}(\Omega)$ *and* $v\in L_{\beta}(\mathbb{R}^{n})\cap\mathcal {C}_{\mathrm{loc}}^{1,1}(\Omega)$ *be positive solutions of the system*

1.3 $$ \textstyle\begin{cases} (-\Delta)^{\frac{\alpha}{2}}u+au=f(v), \quad {x}\in\Omega, \\ (-\Delta)^{\frac{\beta}{2}}v+bv=g(u),\quad {x}\in\Omega, \\ u\geq0,\qquad v\geq0,\quad {x}\in\Omega, \\ u=v=0,\quad {x}\notin\Omega, \end{cases}\displaystyle \quad \textit{for some } a,b\geq0, $$

*with* $M>f'(\cdot)$, $g'(\cdot)>0$, *where* *M* *is a positive constant*. *Then* *u*, *v* *are increasing in* $x_{n}$.

Now we consider the whole space case.

### Theorem 1.3

*Let* $u\in L_{\alpha}(\mathbb{R}^{n})\cap\mathcal {C}_{\mathrm {loc}}^{1,1}(\mathbb{R}^{n}), v \in L_{\beta}(\mathbb{R}^{n})\cap \mathcal {C}_{\mathrm{loc}}^{1,1}(\mathbb{R}^{n})$ *be positive solutions of the system*

1.4 $$ \textstyle\begin{cases} (-\Delta)^{\frac{\alpha}{2}}u+au=f(v),\quad {x}\in\mathbb{R}^{n}, \\ (-\Delta)^{\frac{\beta}{2}}v+bv=g(u),\quad {x}\in\mathbb{R}^{n}, \\ u>0,\qquad v>0, \quad {x}\in\mathbb{R}^{n}, \end{cases}\displaystyle \quad \textit{for some } a,b\geq0. $$

*Suppose that*, *for* $\gamma,\nu>0$,

1.5 $$ u(x)=o\biggl(\frac{1}{|x|^{\nu}}\biggr)\quad \textit{and}\quad v(x)=o\biggl(\frac{1}{|x|^{\gamma}}\biggr)\quad \textit{as } |x|\rightarrow\infty $$

*and*

1.6 $$ 0< f'(s)\leq s^{p} \quad \textit{and}\quad 0< g'(s)\leq s^{q} \quad \textit{with } p\gamma\geq \alpha \textit{ and } q\nu\geq\beta. $$

*Then* $u(x)$ *and* $v(x)$ *are radially symmetric and decreasing about some point* $x_{0}$ *in* $\mathbb{R}^{n}$.

Now we consider the nonexistence of positive solutions to system (1.1) in the half-space.

### Theorem 1.4

*Let* $u\in L_{\alpha}(\mathbb{R}_{+}^{n})\cap\mathcal {C}_{\mathrm{loc}}^{1,1}(\mathbb {R}_{+}^{n})$ *and* $v\in L_{\beta}(\mathbb{R}_{+}^{n})\cap\mathcal {C}_{\mathrm{loc}}^{1,1}(\mathbb{R}_{+}^{n})$ *be nonnegative solutions of the system*

1.7 $$ \textstyle\begin{cases} (-\Delta)^{\frac{\alpha}{2}}u+au=f(v),\quad {x}\in\mathbb{R}_{+}^{n}, \\ (-\Delta)^{\frac{\beta}{2}}v+bv=g(u), \quad {x}\in\mathbb{R}_{+}^{n}, \\ u\equiv0,\qquad v\equiv0,\quad {x}\notin\mathbb{R}_{+}^{n}, \end{cases}\displaystyle \quad \textit{for some } a,b\geq0. $$

*Suppose*

1.8 $$ \varliminf_{|x|\rightarrow\infty}u(x)=0,\qquad \varliminf_{|x|\rightarrow \infty}v(x)=0, $$

*and* $M>f'(\cdot)$, $g'(\cdot)>0$, *where* *M* *is a positive constant with* $f(0)=0$, $g(0)=0$. *Then* $u(x)\equiv0$ *and* $v(x)\equiv0$ *in* $\mathbb{R}^{n}$.

### Remark 1.2

In Sect. 2, we introduce two maximum principles, namely, the narrow region principle and decay at infinity. This two maximum principles play a key role in the proof of Theorems 1.1–1.4. We give detailed proofs of our main theorems in Sect. 3.

## Two maximum principles

Let $T_{\lambda}$ be a hyperplane in $\mathbb{R}^{n}$. Without loss of generality, we assume that

$$T_{\lambda}=\bigl\{ x=\bigl(x_{1},x'\bigr)\in \mathbb{R}^{n}\mid x_{1}=\lambda,\lambda\in \mathbb{R}\bigr\} , $$

where $x'=(x_{2},x_{3},\ldots,x_{n})$. Let

$$x^{\lambda}=(2\lambda-x_{1},x_{2},\ldots,x_{n}) $$

be the reflection of *x* about the plane $T_{\lambda}$. Set

$$\begin{aligned}& \Sigma_{\lambda}= \bigl\{ x\in\mathbb{R}^{n}:x_{1}< \lambda\bigr\} ,\qquad \Sigma_{\lambda}^{c}=\bigl\{ \mathbb{R}^{n}\setminus \Sigma_{\lambda}\bigr\} , \\& u_{\lambda}(x)=u\bigl(x^{\lambda}\bigr),\qquad U_{\lambda}(x)=u_{\lambda}(x)-u(x), \qquad V_{\lambda}(x)=u_{\lambda}(x)-u(x),\quad \forall x\in \mathbb{R}^{n}. \end{aligned}$$

For simplicity of notations, we denote $U_{\lambda}(x)$ by $U(x)$ and $V_{\lambda}(x)$ by $V(x)$.

### Lemma 2.1

(Narrow region principle)

*Let* Ω *be a bounded narrow region in* $\Sigma_{\lambda}$ *that is contained in*

$$\{x\mid\lambda-l< x_{1}< \lambda\} $$

*for small* *l*. *Let* $U, V\in L_{\alpha}(\mathbb{R}^{n})\cap\mathcal {C}_{\mathrm {loc}}^{1,1}(\Omega)$ *and suppose that* *U*, *V* *are lower semicontinuous on* Ω̅. *Assume that* $C_{1}(x)$ *and* $C_{4}(x)$ *are bounded from below in* Ω, *whereas* $C_{2}(x), C_{3}(x)<0$ *are bounded from below in* Ω. *If* *U*, *V* *satisfy the system*

2.1 $$ \textstyle\begin{cases} (-\Delta)^{\frac{\alpha}{2}}U(x)+C_{1}(x)U(x)+C_{2}(x)V(x)\geq{0},\quad {x}\in \Omega, \\ (-\Delta)^{\frac{\beta}{2}}V(x)+C_{3}(x)U(x)+C_{4}(x)V(x)\geq{0},\quad {x}\in \Omega, \\ U(x)\geq{0},\qquad V(x)\geq{0}, \quad {x}\in\Sigma_{\lambda}\setminus\Omega, \\ U(x^{\lambda})=-U(x),\quad {x}\in\Sigma_{\lambda}, \\ V(x^{\lambda})=-V(x), \quad {x}\in\Sigma_{\lambda}, \end{cases} $$

*then*, *for sufficiently small* *l*, *we have*

2.2 $$ U(x)\geq{0},\qquad V(x)\geq{0},\quad {x}\in\Omega. $$

*These conclusions hold for an unbounded domain* Ω *if we further assume that*

$$\begin{aligned} \varliminf_{|x|\rightarrow\infty}U(x)\geq{0} \quad \textit{and}\quad \varliminf_{|x|\rightarrow\infty}V(x)\geq{0}. \end{aligned}$$

*Furthermore*, *if there exists* $x_{0}\in\Omega$ *such that*

$$U(x_{0})=0 \quad \textit{or}\quad V(x_{0})=0, $$

*then*

2.3 $$ U(x)\equiv V(x)\equiv0,\quad x\in\mathbb{R}^{n}. $$

### Remark 2.1

After finish this paper, we have found that this lemma was given by Niu and Wang [23]. For completeness, we give a full proof of the lemma with some changes.

### Proof of Lemma 2.1

Suppose on the contrary, that (2.2) is false. Without loss of generality, we assume that there exists a point such that $U(x)<0$. Since $U(x)$ is lower semicontinuous on Ω̅, there exists $x_{0}\in \Omega$, such that

2.4 $$ U(x_{0})=\min_{\Omega}{U(x)}< 0. $$

Now let $\Sigma_{\lambda}^{c}=\mathbb{R}^{n}\setminus \Sigma_{\lambda}$. Then by the definition of $(-\Delta)^{\frac{\alpha}{2}}$ we have

2.5 $$\begin{aligned} &(-\Delta)^{\frac{\alpha}{2}}U(x_{0}) \\ &\quad =C_{n,\alpha}\operatorname{P.V.} \int_{\mathbb {R}^{n}}\frac{U(x_{0})-U(y)}{ |x_{0}-y|^{n+\alpha}}\,dy \\ &\quad =C_{n,\alpha}\operatorname{P.V.} \int_{\Sigma_{\lambda}}\frac{U(x_{0})-U(y)}{ |x_{0}-y|^{n+\alpha}}\,dy+C_{n,\alpha} \int_{\Sigma_{\lambda}^{c}}\frac {U(x_{0})-U(y)}{|x_{0}-y|^{n+\alpha}}\,dy \\ &\quad =C_{n,\alpha}\operatorname{P.V.} \int_{\Sigma_{\lambda}}\frac{U(x_{0})-U(y)}{ |x_{0}-y|^{n+\alpha}}\,dy+C_{n,\alpha} \int_{\Sigma_{\lambda}}\frac {U(x_{0})+U(y)}{|x_{0}-y^{\lambda}|^{n+\alpha}}\,dy \\ &\quad =C_{n,\alpha}\operatorname{P.V.} \int_{\Sigma_{\lambda}}\biggl[\frac {1}{|x_{0}-y|^{n+\alpha }}-\frac{1}{|x_{0}-y^{\lambda}|^{n+\alpha}}\biggr] \bigl[U(x_{0})-U(y)\bigr]\,dy \\ &\qquad {} +C_{n,\alpha} \int_{\Sigma_{\lambda}}\frac {2U(x_{0})}{|x_{0}-y^{\lambda }|^{n+\alpha}}\,dy \\ &\quad :=I_{1}+I_{2}. \end{aligned}$$

To estimate $I_{1}$, we notice that

2.6 $$ \frac{1}{|x_{0}-y|^{n+\alpha}}>\frac{1}{|x_{0}-y^{\lambda}|^{n+\alpha}} \quad \mbox{for } y\in \Sigma_{\lambda}. $$

Since $U(y)\geq0$ and $y\in\Sigma_{\lambda} \setminus \Omega$, by (2.4) we have

$$U(x_{0})-U(y)\leq0 \quad \mbox{for } y\in\Sigma_{\lambda}. $$

We can see that

$$I_{1}\leq0, $$

which implies

2.7 $$ (-\Delta)^{\frac{\alpha}{2}}U(x_{0})\leq I_{2}=2C_{n,\alpha}U(x_{0}) \int _{\Sigma_{\lambda}}\frac{dy}{|x_{0}-y^{\lambda}|^{n+\alpha}}. $$

Choose $x_{0}^{*}=(3l+(x_{0})_{1},x')$ in $\Sigma_{\lambda}^{c}$, where $x_{0}=((x_{0})_{1},x')$. It is easy to see that $B_{l}(x_{0}^{*})\subset\Sigma _{\lambda}^{c}$. Moreover, there exists $C>0$ such that

$$\begin{aligned} \int_{\Sigma_{\lambda}}\frac{dy}{|x_{0}-y^{\lambda}|^{n+\alpha}}& = \int_{\Sigma_{\lambda}^{c}}\frac{dy}{|x_{0}-y|^{n+\alpha}} \\ &\geq \int_{B_{l}(x_{0}^{*})}\frac{dy}{|x_{0}-y|^{n+\alpha}} \\ &\geq \int_{B_{l}(x_{0}^{*})}\frac{dy}{4^{n+\alpha}l^{n+\alpha}} \\ &=\frac{C}{l^{\alpha}}. \end{aligned}$$

Combining the previous estimate with (2.7), we have

2.8 $$ (-\Delta)^{\frac{\alpha}{2}}U(x_{0})\leq\frac{CU(x_{0})}{l^{\alpha}}. $$

Combining (2.8) with (2.1), we have

$$ \frac{CU(x_{0})}{l^{\alpha}}+C_{1}(x_{0})U(x_{0})+C_{2}(x_{0})V(x_{0}) \geq{0}, $$

which is equivalent to

2.9 $$ U(x_{0})\biggl[\frac{C}{l^{\alpha}}+C_{1}(x_{0}) \biggr]\geq-C_{2}(x_{0})V(x_{0}). $$

Since we can choose *l* small enough and $C_{1}$ is bounded from below, we have $\frac{C}{l^{\alpha}}+C_{1}(x_{0})>0$. Since

2.10 $$ U(x_{0})\geq-\frac{C_{2}(x_{0})}{\frac{C}{l^{\alpha}}+C_{1}(x_{0})}V(x_{0}), $$

by the condition $C_{2}(x)<0$ and (2.4) we get that

$$V(x_{0})< 0. $$

On the other hand, *V* is lower semicontinuous on Ω̅; hence there exists $\overline{x}_{0} $ such that

2.11 $$ V(\overline{x}_{0} )=\min_{\Omega}V(x)< 0. $$

Similarly to (2.8), we derive that

2.12 $$ (-\Delta)^{\frac{\beta}{2}}V(\overline{x}_{0} )\leq \frac{CV(\overline{x}_{0} )}{l^{\beta}}. $$

Combining (2.8) and (2.12) with (2.1), we have

$$\begin{aligned} 0&\leq(-\Delta)^{\frac{\beta}{2}}V(\overline{x}_{0} )+C_{3}( \overline {x_{0}})U(\overline{x}_{0} )+C_{4}( \overline{x_{0}})V(\overline{x}_{0} ) \\ &\leq\frac{CV(\overline{x}_{0} )}{l^{\beta}}+C_{3}(\overline{x}_{0} )U(x_{0})+C_{4}(\overline{x}_{0} )V(\overline{x}_{0} ) \\ &\leq\biggl[\frac{C}{l^{\beta}}+C_{4}(\overline{x}_{0} ) \biggr]V(\overline{x}_{0} ) -C_{3}(\overline{x}_{0} )\frac{C_{2}(x_{0})}{\frac{C}{l^{\alpha}}+C_{1}(x_{0})}V(x_{0}) \\ &\leq\biggl[\frac{C}{l^{\beta}}+C_{4}(\overline{x}_{0} ) \biggr]V(\overline{x}_{0} ) -C_{3}(\overline{x}_{0} )\frac{C_{2}(x_{0})}{\frac{C}{l^{\alpha}}+C_{1}(x_{0})}V(\overline{x}_{0} ) \\ &=V(\overline{x}_{0} )\biggl[\frac{C}{l^{\beta}}+C_{4}( \overline{x}_{0})-C_{3}(\overline {x_{0}}) \frac{C_{2}(x_{0})}{\frac{C}{l^{\alpha}}+C_{1}(x_{0})}\biggr]. \end{aligned}$$

We notice that $C_{4}(x)$ is bounded from below in Ω and $C_{2}(x), C_{3}(x)<0$ are bounded from below in Ω. Choosing *l* small enough, we can derive that $V(\overline{x}_{0})\geq0$. This yields a contradiction with (2.11). So (2.2) holds.

Furthermore, if Ω is an unbounded domain, then by the decay condition of *U*, *V* it is easy to see that the negative minimum of *U*, *V* cannot be taken at infinity.

Now we prove (2.3). Without loss of generality, we assume that there exists $x_{0}\in\Omega$ such that $U(x_{0})=0$. Then, due to (2.2) and the fact that $C_{2}(x_{0})V(x_{0})\leq0$, combining (2.5) with the first equation of (2.1), we have

$$\begin{aligned} 0&\leq(-\Delta)^{\frac{\alpha}{2}}U(x_{0})+C_{2}(x_{0})V(x_{0}) \\ &\leq C_{n,\alpha}\operatorname{P.V.} \int_{\Sigma_{\lambda}}\biggl[\frac {1}{|x_{0}-y|^{n+\alpha }}-\frac{1}{|x_{0}-y^{\lambda}|^{n+\alpha}}\biggr] \bigl[-U(y)\bigr]\,dy. \end{aligned}$$

If $U(y)\not\equiv0$, $y\in\Sigma_{\lambda}$, then noticing that $U(y)\geq 0$, $y\in\Sigma_{\lambda}$, we have

$$ (-\Delta)^{\frac{\alpha}{2}}U(x_{0})+C_{2}(x_{0})V(x_{0})< 0. $$

This yields a contradiction. So

$$U(y)\equiv0,\quad y\in\Sigma_{\lambda}. $$

By (2.10) we immediately get $V(x)\equiv0, x\in\Sigma_{\lambda}$. So since *U* and *V* are antisymmetric functions, we have (2.3). □

### Lemma 2.2

(Decay at infinity)

*Let* Ω *be an unbounded domain in* $\Sigma_{\lambda}$. *Let* $U, V\in L_{\alpha}(\mathbb{R}^{n}) \cap C_{\mathrm{loc}}^{1,1}(\Omega)$ *be lower semicontinuous on* Ω̅. *Assume that*

2.13 $$ \textstyle\begin{cases} (-\Delta)^{\frac{\alpha}{2}}U(x)+C_{1}(x)U(x)+C_{2}(x)V(x)\geq{0},\quad {x}\in \Omega, \\ (-\Delta)^{\frac{\beta}{2}}V(x)+C_{3}(x)U(x)+C_{4}(x)V(x)\geq{0},\quad {x}\in \Omega, \\ U(x)\geq{0},\qquad V(x)\geq{0}, \quad {x}\in\Sigma_{\lambda}\setminus\Omega, \\ U(x^{\lambda})=-U(x),\quad {x}\in\Sigma_{\lambda}, \\ V(x^{\lambda})=-V(x),\quad {x}\in\Sigma_{\lambda}, \end{cases} $$

*where* $C_{1}(x)$, $C_{4}(x)$ *are nonnegative on* Ω, *whereas* $C_{2}(x),C_{3}(x)<0$ *on* Ω, *and furthermore*,

2.14 $$ \varliminf_{|x|\rightarrow\infty}C_{2}(x)|x|^{\alpha}=0 \quad \textit{and}\quad \varliminf_{|x|\rightarrow\infty}C_{3}(x)|x|^{\beta}=0. $$

*Then there exists a constant* $R_{0}>0$ (*depending on* $C_{i}(x)$ *but independent of* *U*, *V*) *such that if*

$$U(\widetilde{x})=\min_{\overline{\Omega}}{U(x)}< 0\quad \textit{and}\quad V( \overline{x})=\min_{\overline{\Omega}} V(x)< 0, $$

*then*

$$\widetilde{x}\leq R_{0} \quad \textit{or}\quad \overline{x}\leq R_{0}. $$

### Remark 2.2

This lemma is quite the same as that of Niu and Wang [23] but with some difference. In [23], $C_{1}$, $C_{4}$ should satisfying

2.15 $$ \varliminf_{|x|\rightarrow\infty}C_{1}(x)|x|^{\alpha}=0 \quad \text{and}\quad \varliminf_{|x|\rightarrow\infty}C_{4}(x)|x|^{\beta}=0. $$

So we conclude that the condition of our lemma is different with [23], even they both have the same result.

### Proof of Lemma 2.2

Without loss of generality, we assume that there exist a point $x_{0}\in \Omega$ such that

$$U(\widetilde{x})=\min_{\Omega}{U(x)}< 0. $$

Then as in the proof as (2.7), we have

2.16 $$ (-\Delta)^{\frac{\alpha}{2}}U(\widetilde{x})\leq I_{2}=C_{n,\alpha } \int _{\Sigma_{\lambda_{0}}}\frac{2U(\widetilde{x})}{|\widetilde {x}-y^{\lambda }|^{n+\alpha}}\,dy. $$

Choose a point in $\Sigma_{\lambda}^{c}:\widetilde{x}^{\ast }=(3|\widetilde {x}|+x_{1},x')$, where $\widetilde{x}=(x_{1},x')$. Then $B_{|\widetilde {x}|}(\widetilde{x}^{\ast})\subset\Sigma_{\lambda}^{c}$, and there exists $C>0$ such that

2.17 $$ \int_{\Sigma_{\lambda}}\frac{1}{|\widetilde{x}-y^{\lambda }|^{n+\alpha }}\,dy= \int_{\Sigma_{\lambda}^{c}}\frac{1}{|\widetilde{x}-y|^{n+\alpha}}\,dy \geq \int_{B_{|\widetilde{x}|}(\widetilde{x}^{\ast})}\frac {1}{|\widetilde {x}-y|^{n+\alpha}}\,dy\geq\frac{C}{|\widetilde{x}|^{\alpha}}. $$

So combining (2.17) with (2.16), we have

2.18 $$ (-\Delta)^{\frac{\alpha}{2}}U(\widetilde{x})\leq\frac {CU(\widetilde {x})}{|\widetilde{x}|^{\alpha}}. $$

By (2.13) we have that

$$ (-\Delta)^{\frac{\alpha}{2}}U(\widetilde{x})+C_{1}(\widetilde {x})U( \widetilde{x})+C_{2}(\widetilde{x})V(\widetilde{x})\geq{0}. $$

Combining this with (2.18), we get that

$$ \frac{CU(\widetilde{x})}{|\widetilde{x}|^{\alpha}}+C_{1}(\widetilde {x})U(\widetilde{x}) \geq-C_{2}(\widetilde{x})V(\widetilde{x}). $$

Now by the conditions $C_{1}(\widetilde{x})\geq0$ and $C_{2}(\widetilde {x})<0$ we easily calculate that

2.19 $$ U(\widetilde{x})\geq-\frac{C_{2}(\widetilde{x})}{\frac {C}{|\widetilde {x}|^{\alpha}+C_{1}(\widetilde{x})}}V(\widetilde{x}). $$

Noticing that $U(\widetilde{x})<0$ and $C_{2}(\widetilde{x})<0$, we get $V(\widetilde{x})<0$. Then since *V* are lower semicontinuous on Ω̅. there exists $\overline{x_{0}}$ such that

2.20 $$ V(\overline{x})=\min_{\Omega}V(x)< 0. $$

Similarly to (2.18), we derive that

2.21 $$ (-\Delta)^{\frac{\beta}{2}}V(\overline{x})\leq\frac{CV(\overline{x})}{|\overline{x}|^{\beta}}. $$

Combining (2.19) and (2.21) with (2.13), we have

$$\begin{aligned} 0&\leq(-\Delta)^{\frac{\beta}{2}}V(\overline{x})+C_{3}(\overline{x})U( \overline{x})+C_{4}(\overline{x})V(\overline{x}) \\ &\leq\frac{CV(\overline{x})}{|\overline{x}|^{\beta}}+C_{3}(\overline{x})U(\widetilde{x})+C_{4}( \overline{x})V(\overline{x}) \\ &\leq\biggl[\frac{C}{|\overline{x}|^{\beta}}+C_{4}(\overline{x})\biggr]V(\overline{x}) -C_{3}(\overline{x})\frac{C_{2}(\widetilde{x})}{\frac{C}{|\widetilde {x}|^{\alpha}}+C_{1}(\widetilde{x})}V(\widetilde{x}) \\ &\leq\biggl[\frac{C}{|\overline{x}|^{\beta}}+C_{4}(\overline{x})\biggr]V(\overline{x}) -C_{3}(\overline{x})\frac{C_{2}(\widetilde{x})}{\frac{C}{|\widetilde {x}|^{\alpha}}+C_{1}(\widetilde{x})}V(\overline{x}) \\ &=V(\overline{x})\biggl[\frac{C}{|\overline{x}|^{\beta}}+C_{4}(\overline{x})-C_{3}(\overline{x} )\frac{C_{2}(\widetilde{x})}{\frac{C}{|\widetilde {x}|^{\alpha}}+C_{1}(\widetilde{x})}\biggr]. \end{aligned}$$

Choosing $|\widetilde{x}|$, $|\overline{x}|$ large enough, by (2.14) we can derive $V(\overline{x})\geq0$. This yields a contradiction with (2.20). So the lemma is proved. □

## Proof of Theorems 1.1-1.4

### Proof of Theorem 1.1

Let $T_{\lambda}$, $x_{\lambda}$, $u_{\lambda}$, $\Sigma_{\lambda}$, and $U_{\lambda}$, $V_{\lambda}$ be defined as in the previous section.

Step 1: We will show that for $\lambda>-1$ and sufficiently close to −1, we have

3.1 $$ U_{\lambda}(x)\geq0,\qquad V_{\lambda}(x)\geq0,\quad x \in\Sigma_{\lambda}\cap B. $$

By the first equation in system (1.2) and the mean value theorem it is easy to see that

3.2 $$ (-\Delta)^{\frac{\alpha}{2}}U_{\lambda}(x)+aU_{\lambda}(x)-f' \bigl(\xi (x)\bigr)V_{\lambda}(x)=0, $$

where $\xi(x)$ is between $v(x)$ and $v_{\lambda}(x)$. Similarly, we also have

$$ (-\Delta)^{\frac{\beta}{2}}V_{\lambda}(x)+bV_{\lambda}(x)-g' \bigl(\eta (x)\bigr)U_{\lambda}(x)=0, $$

where $\eta(x)$ is between $u(x)$ and $u_{\lambda}(x)$. Choosing $C_{1}=a$, $C_{2}=-f'(\xi(x))$, $C_{3}=b$, and $C_{4}=-g'(\eta(x))$, by the narrow region principle (Lemma 2.1) we get (3.1).

Step 2: Define

$$\lambda_{0}=\sup\bigl\{ \lambda\leq0\mid U_{\mu}(x) \geq0,V_{\mu}(x)\geq0, \forall x\in \Sigma_{\mu},\forall\mu\leq \lambda\bigr\} . $$

Then we claim that

3.3 $$ \lambda_{0}=0. $$

Suppose the claim is not true. If $\lambda_{0} < 0$, then we will show that the plane can be moved to the right a little more so that inequality (3.1) will still valid. More precisely, there exists small $\epsilon> 0$ such that, for all $\lambda\in[\lambda_{0},\lambda_{0}+\epsilon)$, inequality (3.1) holds, which contradicts the definition of $\lambda_{0}$.

First, since $U_{\lambda_{0}}$ and $V_{\lambda_{0}}$ are not identically zero, from the proof of the narrow region principle (Lemma 2.1) we have

$$U_{\lambda_{0}}>0,\qquad V_{\lambda_{0}}>0,\quad \forall x\in \Sigma_{\lambda _{0}}\cap B. $$

Thus $U_{\lambda_{0}}$ and $V_{\lambda_{0}}$ can take the minimum values if $x\in\Sigma_{\lambda_{0}-\delta}\cap B$. More precisely, for any $\delta>0$,

$$U_{\lambda_{0}}\geq c_{\delta}>0,\qquad V_{\lambda_{0}}\geq c_{\delta}>0,\quad \forall x\in \Sigma_{\lambda_{0}-\delta}\cap B. $$

By the continuity of $U_{\lambda}$ and $V_{\lambda}$ with respect to *λ* there exists $\epsilon> 0$ such that

$$U_{\lambda}\geq0,\qquad V_{\lambda}\geq0,\quad \forall x\in \Sigma_{\lambda _{0}-\delta }\cap B, \forall\lambda\in[\lambda_{0}, \lambda_{0}+\epsilon). $$

We can see that $(\Sigma_{\lambda} \setminus \Sigma_{\lambda_{0}-\delta })\cap B$ is a narrow region if *ϵ* and *δ* are small enough. Then by the narrow region principle (Lemma 2.1) we have

$$U_{\lambda}\geq0,\qquad V_{\lambda}\geq0,\quad \forall x\in \Sigma_{\lambda}\cap B, \forall\lambda\in[\lambda_{0}, \lambda_{0}+\epsilon). $$

This contradicts the definition of $\lambda_{0}$. Therefore we prove the claim (3.3). It follows that

$$U_{0}\geq0,\qquad V_{0}\geq0,\quad x\in \Sigma_{0}\cap B, $$

or, more apparently,

$$u\bigl(-x_{1},x'\bigr)\leq u\bigl(x_{1},x' \bigr),\qquad v\bigl(-x_{1},x'\bigr)\leq v \bigl(x_{1},x'\bigr), \quad 0< x_{1}< 1. $$

Since the $x_{1}$-direction can be chosen arbitrarily, it follows that $u,v$ are radially symmetric about the origin. The monotonicity is a consequence of the fact that

$$U_{\lambda}\geq0,\qquad V_{\lambda}\geq0,\quad x\in \Sigma_{\lambda}, $$

for all $-1<\lambda\leq0$. This completes the proof of Theorem 1.1. □

### Proof of Theorem 1.2

Let $T_{\lambda}$, $x_{\lambda}$, $u_{\lambda}$, and $U_{\lambda}$, $V_{\lambda}$ be defined as in the previous section. Let

$$\Sigma_{\lambda}=\bigl\{ x=\bigl(x',x_{n}\bigr)\mid x_{n}< \lambda\bigr\} . $$

Step 1: Similarly to (3.1), we can get that, for $\lambda>0$ sufficiently close to 0, we have

3.4 $$ U_{\lambda}(x)\geq0, \qquad V_{\lambda}(x)\geq0,\quad x \in\Sigma_{\lambda}\cap \Omega. $$

Step 2: Define

$$\lambda_{0}=\sup\bigl\{ \lambda>0\mid U_{\mu}(x) \geq0,V_{\mu}(x)\geq0, \forall x\in \Sigma_{\mu},\forall\mu\leq \lambda\bigr\} . $$

Then we must have

$$\lambda_{0}=+\infty. $$

Otherwise, suppose that $\lambda_{0} <+\infty$, Then we claim that

3.5 $$ U_{\lambda_{0}}(x)\equiv0,\qquad V_{\lambda_{0}}(x)\equiv0, \quad x\in\Sigma _{\lambda _{0}}\cap\Omega. $$

If (3.5) were not true, then by the narrow region principle (Lemma 2.1) we would have

3.6 $$ U_{\lambda_{0}}(x)>0,\qquad V_{\lambda_{0}}(x)>0,\quad x\in \Sigma_{\lambda_{0}}\cap \Omega. $$

We will show that the plane $T_{\lambda}$ can be moved further to the right. More precisely, there exists small $\epsilon>0$ such that, for all $\lambda\in[\lambda_{0},\lambda_{0}+\epsilon)$,

3.7 $$ U_{\lambda}(x)\geq0,\qquad V_{\lambda}(x)\geq0,\quad x \in\Sigma_{\lambda}\cap \Omega. $$

This is a contraction with the definition of $\lambda_{0}$.

If (3.6) holds, then for any $\delta>0$,

$$U_{\lambda_{0}}\geq c_{\delta}>0,\qquad V_{\lambda_{0}}\geq c_{\delta}>0,\quad \forall x\in \Sigma_{\lambda_{0}-\delta}\cap\Omega. $$

By the continuity of $U_{\lambda}$, $V_{\lambda}$ with respect to *λ* there exists $\epsilon> 0$ such that

$$U_{\lambda}\geq0,\qquad V_{\lambda}\geq0,\quad \forall x\in \Sigma_{\lambda _{0}-\delta }\cap\Omega, \forall\lambda\in[\lambda_{0}, \lambda_{0}+\epsilon). $$

We can see that $\Sigma_{\lambda} \setminus \Sigma_{\lambda_{0}-\delta }$ is a narrow region since *ϵ* and *δ* are small enough. Then by the narrow region principle (Lemma 2.1) we have

$$U_{\lambda}\geq0,\qquad V_{\lambda}\geq0, \quad \forall x\in \Sigma_{\lambda}\cap\Omega , \forall\lambda\in[\lambda_{0}, \lambda_{0}+\epsilon). $$

This contradicts the definition of $\lambda_{0}$. Then the claim (3.5) holds, which implies

$$u\bigl(x',2\lambda_{0}\bigr)=u\bigl(x',0 \bigr)=0, \qquad v\bigl(x',2\lambda_{0}\bigr)=v \bigl(x',0\bigr)=0, $$

which contradicts the fact that $u,v>0$ on Ω. We have shown that $\lambda_{0}=\infty$ and $U\geq0$, $V\geq0$. This shows that $u(x)$ and $v(x)$ are increasing in $x_{n}$, which completes the proof of Theorem 1.2. □

### Proof of Theorem 1.3

Start moving the plane $T_{\lambda}$ from −∞ to that right along the $x_{1}$-direction.

Step 1: We will show that for *λ* sufficiently negative,

3.8 $$ U_{\lambda}(x)\geq0,\qquad V_{\lambda}(x)\geq0,\quad x \in\Sigma_{\lambda}. $$

By the first equation in system (1.4) and the mean value theorem it is easy to see that

3.9 $$ (-\Delta)^{\frac{\alpha}{2}}U_{\lambda}(x)+aU_{\lambda}(x)-f' \bigl(\xi (x)\bigr)V_{\lambda}(x)=0, $$

where $\xi(x)$ is between $v(x)$ and $v_{\lambda}(x)$. Similarly, we also have

$$ (-\Delta)^{\frac{\beta}{2}}V_{\lambda}(x)+bV_{\lambda}(x)-g' \bigl(\eta (x)\bigr)U_{\lambda}(x)=0, $$

where $\eta(x)$ is between $u(x)$ and $u_{\lambda}(x)$. In fact, $0< v_{\lambda}(x)\leq\xi(x)\leq v(x)$ and $0< u_{\lambda}(x)\leq\eta (x)\leq u(x)$.

At those points, for $|x|$ sufficiently large, the decay assumptions (1.5) and (1.6) immediately yield

$$\begin{aligned} &\varliminf_{|x|\rightarrow\infty}f'\bigl(\xi(x)\bigr)|x|^{\alpha}\\ &\quad \leq\varliminf_{|x|\rightarrow\infty}\xi^{p}(x)|x|^{\alpha}\\ &\quad \leq\varliminf_{|x|\rightarrow\infty}u^{p}(x)|x|^{\alpha}\\ &\quad =0, \end{aligned}$$

and going through a similar proof, we have

$$\varliminf_{|x|\rightarrow\infty}g'\bigl(\eta(x)\bigr)|x|^{\beta}=0. $$

Then by the decay at infinity (Lemma 2.2) there exists a constant $R_{0}>0$ such that for $\lambda<-R_{0}$ in Lemma 2.2, one of $U_{\lambda}(x)$ and $V_{\lambda}(x)$ must be positive in $\Sigma _{\lambda}$. Without loss of generality, we assume that

$$ V_{\lambda}(x)\geq0,\quad x\in\Sigma_{\lambda}. $$

Now we can also prove $U_{\lambda}(x)\geq0$, $x\in\Sigma_{\lambda}$. If not, then by the decay condition of $u(x)$ there must exist a point $x_{0}\in \Sigma_{\lambda}$ such that

3.10 $$ U_{\lambda}( x_{0})=\min_{x\in\Sigma_{\lambda}} U_{\lambda}(x)< 0. $$

From previous arguments (2.18) and (3.9) we have

$$ \frac{CU_{\lambda}(x_{0})}{|x_{0}|^{\alpha}}+aU_{\lambda}(x) -f'\bigl(\xi (x_{0})\bigr)V_{\lambda}(x_{0})\geq0, $$

and then

$$ \biggl(\frac{C}{|x_{0}|^{\alpha}}+a\biggr)U_{\lambda}(x_{0}) \geq f'\bigl(\xi(x_{0})\bigr)V_{\lambda}(x_{0})\geq0, $$

since $f'(\cdot)>0$, $V_{\lambda}(x_{0})\geq0$. We can derive that $U_{\lambda}(x_{0})\geq0$, which contradicts with (3.10). So (3.8) holds.

Step 2: Keep moving the planes to the right to the limiting positive $T_{\lambda_{0}}$ as long as (3.8) holds.

Let

$$\lambda_{0}=\sup\bigl\{ \lambda\mid U_{\mu}(x) \geq0,V_{\mu}(x)\geq0, \forall x\in\Sigma _{\mu},\forall\mu\leq \lambda\bigr\} . $$

We have that

$$\lambda_{0}< \infty. $$

Otherwise, if $\lambda_{0}=\infty$, then the solution $u(x)$ is increasing with respect to $x_{1}$. This contradicts condition (1.5), so that $\lambda_{0}<\infty$.

Then we claim that

3.11 $$ U_{\lambda_{0}}(x)\equiv0,\qquad V_{\lambda_{0}}(x)\equiv0,\quad x\in\Sigma _{\lambda_{0}}. $$

If (3.11) were not true, then by the proof of the narrow region principle (Lemma 2.1) we would have

3.12 $$ U_{\lambda_{0}}(x)>0,\qquad V_{\lambda_{0}}(x)>0,\quad x\in \Sigma_{\lambda_{0}}. $$

We will show that the plane $T_{\lambda}$ can be moved further to the right. More precisely, there exists small $\epsilon>0$ such that, for all $\lambda\in[\lambda_{0},\lambda_{0}+\epsilon)$,

3.13 $$ U_{\lambda}(x)\geq0,\qquad V_{\lambda}(x)\geq0,\quad x \in\Sigma_{\lambda}. $$

This is a contraction with the definition of $\lambda_{0}$.

If (3.12) is true, then let $R_{0}$ be determined in the decay at infinity (Lemma 2.2). It follows that, for any $\delta>0$,

$$U_{\lambda_{0}}\geq C_{0}>0,\qquad V_{\lambda_{0}}(x)\geq C_{0}>0, \quad x\in\overline {\Sigma _{\lambda_{0}-\delta}\cap B_{R_{0}}(0)}. $$

Since we have the continuity of $U_{\lambda}(x)$ and $V_{\lambda}(x)$ with respect to *λ*, there exists $\epsilon>0$ such that, for any $\lambda\in[\lambda_{0},\lambda+\epsilon)$,

3.14 $$ U_{\lambda}(x)\geq0,\qquad V_{\lambda}(x)\geq0,\quad x \in\overline{\Sigma _{\lambda _{0}-\delta}\cap B_{R_{0}}(0)}. $$

Suppose (3.13) is not true. If $x_{0}$ and $\overline{x_{0}}$ are the negative minima of $U_{\lambda}(x)$ and $V_{\lambda}(x)$ in $\Sigma _{\lambda}$, then by decay at infinity (Lemma 2.2) and (3.14) we can get that they are all in the bounded narrow region $(\Sigma_{\lambda_{0}+\epsilon} \setminus \Sigma_{\lambda_{0}-\delta })\cap B_{R_{0}}(0)$ for *δ* and *ϵ* small enough, which contradicts the narrow region principle (Lemma 2.1). So (3.13) has to be true, which is a contradiction with the definition of $\lambda_{0}$.

Now we have proved the claim (3.11). Since the $x_{1}$-direction can be chosen arbitrarily, we get that $u(x)$ and $v(x)$ are radially symmetric and decreasing about some point $x_{0}$. This completes the proof of Theorem 1.3. □

### Proof of Theorem 1.4

First, we claim that

3.15 $$ u(x)>0,\qquad v(x)>0,\quad x\in\mathbb{R}^{n}_{+}\quad \mbox{or}\quad u(x)\equiv0,\qquad v(x)\equiv0,\quad x\in \mathbb{R}^{n}_{+}. $$

To prove (3.15), we assume that $u(x)\not\equiv0$. If there exists $x_{0}\in\mathbb{R}^{n}_{+} $ such that $u(x_{0})=0$, then we have that

$$\begin{aligned} (-\Delta)^{\frac{\alpha}{2}}u(x_{0})& = C_{n,\alpha} \operatorname{P.V.} \int_{\mathbb{R}^{n}} \frac {u(x_{0})-u(y)}{|x_{0}-y|^{n+\alpha}} \,dy \\ &=-C_{n,\alpha}\operatorname{P.V.} \int_{\mathbb{R}^{n}}\frac{u(y)}{|x_{0}-y|^{n+\alpha }}\,dy \\ &< 0. \end{aligned}$$

On the other hand, by the function (1.7) and the condition on $f(x)$ and $g(x)$ we have that $(-\Delta)^{\frac{\alpha }{2}}u=f(v)-mv\geq0$. This yields a contradiction, so we have that

$$\text{either}\quad u\equiv0\quad \text{or} \quad u>0\quad \text{in } \mathbb{R}^{n}_{+}. $$

If $u\equiv0$ in $\mathbb{R}^{n}_{+}$, by (1.7) we have $f(v(x))\equiv mv$ in $\mathbb{R}^{n}_{+}$. Together with the condition $f'(s)> m$ and $f(0)=0$, we can obtain that $v\equiv0$ in $\mathbb {R}^{n}_{+}$. Hence if $u(x)$ attains 0 somewhere in $\mathbb{R}^{n}_{+}$, then $u(x)=v(x)\equiv0$. Similarly, we can also derive that if $v(x)$ attains 0 somewhere in $\mathbb{R}^{n}_{+}$, then $u(x)=v(x)\equiv0$, so (3.15) holds. Now we assume that

3.16 $$ u(x)>0,\qquad v(x)>0, \quad x\in\mathbb{R}^{n}_{+}. $$

Denote $T_{\lambda}=\{x\in\mathbb{R}^{n}_{+}\mid x_{n}=\lambda,\lambda>0\} ,\Sigma _{\lambda}=\{x\in\mathbb{R}^{n}_{+}\mid 0< x_{n}<\lambda\}$. Let $x^{\lambda}=(x_{1},\ldots,x_{n-1},2\lambda-x_{n})$ be the reflection of *x* about the plane $T_{\lambda}$, and let $U_{\lambda}(x)=u_{\lambda}(x)-u(x)$ and $V_{\lambda}(x)=v_{\lambda}(x)-v(x)$.

Step 1. For $\lambda>0$ sufficiently close to 0, set $\Sigma=\Sigma _{\lambda}\cup\mathbb{R}^{n}_{-}$, where $\mathbb{R}^{n}_{-}=\{x\in\mathbb {R}^{n}\mid x_{n}\leq0\}$.

First, we know that $U(x)=u_{\lambda}(x)-u(x)\geq-u(x)$ and $V(x)=v_{\lambda}(x)-v(x)\geq-v(x)$, so by condition (1.8) we have

$$\lim_{|x|\rightarrow\infty}U_{\lambda}(x)\geq\lim_{|x|\rightarrow \infty }-u(x)=0; \qquad \lim_{|x|\rightarrow\infty}V_{\lambda}(x)\geq\lim _{|x|\rightarrow \infty}-v(x)=0. $$

Then by the narrow region principle (Lemma 2.1) it is easy to see that

3.17 $$ U_{\lambda}(x)\geq0,\qquad V_{\lambda}(x)\geq0,\quad x \in\Sigma_{\lambda}, $$

since $\Sigma_{\lambda}$ is a narrow region.

Step 2. Next, we move the plane $T_{\lambda}$ along the $x_{n}$-axis to the right as long as (3.17) holds and set

$$\lambda_{0}=\sup\bigl\{ \lambda>0\mid U_{\mu}(x) \geq0,V_{\mu}(x)\geq0,\forall x\in\Sigma _{\mu},\forall\mu\leq \lambda\bigr\} . $$

We claim that

$$\lambda_{0}=\infty. $$

Otherwise, if $\lambda_{0}<\infty$, then by the proof of the narrow region principle (Lemma 2.1) we have

3.18 $$ U_{\lambda_{0}}>0,\qquad V_{\lambda_{0}}>0,\quad x\in \Sigma_{\lambda_{0}} \quad \mbox{or}\quad U_{\lambda _{0}}\equiv0,\qquad V_{\lambda_{0}}\equiv0,\quad x\in\Sigma_{\lambda_{0}}. $$

Then going through similar arguments as in (3.11), we can see that

$$U_{\lambda_{0}}\equiv0,\qquad V_{\lambda_{0}}\equiv0,\quad x\in \Sigma_{\lambda_{0}}. $$

If we choose the point $\bar{x}=(x_{1},x_{2},\ldots,x_{n-1},0) $ in the hyperplane $\{x_{n}=0\}$, then $\bar{x}^{\lambda_{0}}\in\mathbb {R}^{n}_{+}$, which implies

$$u(x_{1},x_{2},\ldots,x_{n-1},2\lambda_{0})=u(x_{1},x_{2}, \ldots,x_{n-1},0)=0 $$

and

$$v(x_{1},x_{2},\ldots,x_{n-1},2\lambda_{0})=v(x_{1},x_{2}, \ldots,x_{n-1},0)=0. $$

This contradicts with (3.16).

Therefore we have proved the claim $\lambda_{0}=\infty$, and consequently the solutions $u(x)$ and $v(x)$ are increasing with respect to $x_{n}$. We recall that condition (1.8) tells us that $\varliminf_{|x|\rightarrow\infty}u(x)=0$ and $\varliminf_{|x|\rightarrow\infty}v(x)=0$. So the claim (3.16) is not true, and thus $u(x)\equiv0$ and $v(x)\equiv0$, $x\in\mathbb{R}^{n}$. □
